# Efficacy of front-line immunochemotherapy for follicular lymphoma: a network meta-analysis of randomized controlled trials

**DOI:** 10.1038/s41408-021-00598-x

**Published:** 2022-01-05

**Authors:** Yucai Wang, Shouhao Zhou, Xinyue Qi, Fang Yang, Matthew J. Maurer, Thomas M. Habermann, Thomas E. Witzig, Michael L. Wang, Grzegorz S. Nowakowski

**Affiliations:** 1 Division of Hematology, Mayo Clinic, Rochester, MN USA; 2 Department of Public Health Sciences, Penn State College of Medicine, Hershey, PA USA; 3 Department of Health Services Research, The University of Texas MD Anderson Cancer Center, Houston, TX USA; 4 The Comprehensive Cancer Centre of Drum Tower Hospital, Clinical Cancer Institute of Nanjing University, Nanjing, China; 5 Department of Quantitative Health Sciences, Mayo Clinic, Rochester, MN USA; 6 Department of Lymphoma/Myeloma, The University of Texas MD Anderson Cancer Center, Houston, TX USA

**Keywords:** B-cell lymphoma, Medical research

## Abstract

Front-line treatment for follicular lymphoma has evolved with the introduction of maintenance therapy, bendamustine (Benda), obinutuzumab (G), and lenalidomide (Len). We conducted a random-effects Bayesian network meta-analysis (NMA) of phase 3 randomized controlled trials (RCTs) to identify the regimens with superior efficacy. Progression-free survival (PFS) was compared between 11 modern regimens with different immunochemotherapy and maintenance strategies. G-Benda-G resulted in with the best PFS, with an HR of 0.41 compared to R-Benda, a surface under the cumulative ranking curve (SUCRA) of 0.97, a probability of being the best treatment (PbBT) of 72%, and a posterior ranking distribution (PoRa) of 1 (95% BCI 1–3). This was followed by R-Benda-R4 (HR = 0.49, PbBT = 25%, PoRa = 2) and R-Benda-R (HR = 0.60, PbBT = 3%, PoRa = 3). R-CHOP-R (HR = 0.96) and R-Len-R (HR = 0.97) had similar efficacy to R-Benda. Bendamustine was a better chemotherapy backbone than CHOP either with maintenance (R-Benda-R vs R-CHOP-R, HR = 0.62; G-Benda-G vs G-CHOP-G, HR = 0.55) or without maintenance therapy (R-Benda vs R-CHOP, HR = 0.68). Rituximab maintenance improved PFS following R-CHOP (R-CHOP-R vs R-CHOP, HR = 0.65) or R-Benda (R-Benda-R vs R-Benda, HR = 0.60; R-Benda-R4 vs R-Benda, HR = 0.49). In the absence of multi-arm RCTs that include all common regimens, this NMA provides an important and useful guide to inform treatment decisions.

## Introduction

Follicular lymphoma (FL) is the second most common non-Hodgkin lymphoma in the United States and Europe [1, 2]. The clinical presentation of FL is highly heterogeneous, with different histological grades, clinical stages, and varying degrees of symptoms and cytopenias. In a subset of patients, treatment can be deferred until symptoms arise or certain criteria are met [3]. Radiation therapy and/or anti-CD20 monoclonal antibody (e.g., rituximab) are among appropriate options for early-stage or low bulk disease [4]. Anti-CD20 antibody-based immunochemotherapy is often indicated for advanced-stage disease [5, 6].

There have been significant advances in the front-line treatment of advanced-stage FL in the last decade. The FOLL05 trial established that R-CHOP was superior to R-CVP in terms of progression-free survival (PFS) [7, 8], and the PRIMA trial demonstrated that rituximab maintenance improved PFS following R-CHOP or R-CVP [9, 10]. Rituximab in combination with bendamustine (R-Benda) emerged as a preferred regimen after the StiL NHL1 trial and the BRIGHT trial. Stil NHL1 trial reported improved PFS with R-Benda vs R-CHOP (without maintenance) [11], and BRIGHT trial showed improved PFS with R-Benda vs R-CHOP/R-CVP, with rituximab maintenance allowed in both arms [12–14]. The benefit of rituximab maintenance after R-Benda is unclear, although the StiL NHL7 trial is addressing 2 vs 4 years of rituximab maintenance following R-Benda [15]. More recently, the GALLIUM trial demonstrated the superiority of obinutuzumab vs rituximab [16, 17], and the RELEVANCE trial suggested that rituximab in combination with lenalidomide (R-Len) was also an active regimen [18].

The different options of chemotherapy backbone, anti-CD20 antibody and maintenance therapy are all supported by NCCN and ESMO guidelines [6, 19]. However, there is a need to comprehensively compare the efficacy of modern front-line treatment options. While R-Benda results in favorable PFS in general, patients with high-risk diseases, such as those with a high-risk prognostic index [20–23], may need more efficacious front-line treatment. In addition, patients who experience disease progression within 24 months (POD24) of initial therapy have a poor prognosis [24–27]. Therefore, better front-line therapy is needed in select patient groups. In this study, we conducted a network meta-analysis of randomized controlled trials (RCT) to compare the efficacy of common immunochemotherapy regimens and to inform clinical practice.

## Methods

### Search methods and study selection

This study followed the Preferred Reporting Items for Systematic Reviews and Meta-analysis (PRISMA-NMA) guideline and was registered with PROSPERO (registration number CRD42019133481). A systematic search of the literature was conducted to identify published clinical trials of front-line immunochemotherapy for FL. The search was done in PubMed and Web of Science with the searching terms follicular lymphoma, newly diagnosed, untreated, first-line, front-line, initial, and maintenance. Studies published online ahead of print were eligible. The references of relevant published trials and review articles were also searched for additional eligible studies. Finally, major international meeting abstracts were searched for updated data of potentially eligible studies. The initial search was conducted in January 2019, and a final search for updates was conducted on March 15, 2021. Studies eligible for inclusion met all following criteria: (1) RCT design; (2) provided data on PFS; and (3) published in English. The literature search and study selection were performed independently by two investigators (FY and YW), with discrepancies reviewed and resolved by consensus.

### Data extraction

The study name, phase, inclusion and exclusion criteria, stage, grade, treatment arms, patient number, and treatment effect (hazard ratios [HRs] and 95% confidence interval [CI] for PFS) were extracted from each included study. Two authors extracted data independently (FY and YW), and discrepancies were reviewed and resolved by consensus.

### Network meta-analysis

Network meta-analysis synthesizes the relative effectiveness of multiple treatment arms by pooling direct and indirect evidence from a set of randomized trials [28]. To incorporate direct and indirect comparisons of relative treatment effects, we applied a Bayesian hierarchical model for network meta-analysis and adjusted the random-effects for between-study heterogeneity. The primary endpoint of the network meta-analysis is PFS, which is defined as the time from study entry or randomization or treatment start to disease progression, relapse, or death from any cause.

To prepare for meta-analysis and network comparison, we converted the PFS data by the natural log transformations of HRs and their 95% CI to estimate standard errors. A normal distribution of the log-HR point estimate was assumed across studies, with the mean parameter equal to the contrast of relative treatment effects of relevant interventions. For data with HRs to compare combined treatments, the linear terms in contrast to relative effects were weighted by the sample subgroup proportions. The log-HR variance parameter followed a conjugate inverse gamma distribution, with the shape and scale parameters estimated using the same approach as in the random variance model [29]. A noninformative prior distribution was proposed for the mean parameters of normal distributions for relative treatment effects and study effects, and half-Cauchy prior distribution with the mode at 0 and scale at 2.5 was proposed for the standard deviation parameters [30, 31]. To investigate the impact of the weakly informative Cauchy prior, we compared the results with a noninformative uniform prior on (0,100) and half-Cauchy prior with different scale parameters. To examine the robustness of the statistical inference given the adopted inclusion criteria, we also conducted the following three sensitivity analyses: (i) excluding the data on the treatment effect estimation between R-Benda and R-Benda-R from the cross-trial comparison in Stil NHL1 and NHL7 studies; (ii) from (i), further excluding the data on the post hoc assessment of treatment effects for maintenance therapy in the BRIGHT trial; and (iii) from (ii), further excluding the treatment effect estimation with the randomized but combined control group data in the BRIGHT study, to assess their influence on the results.

Because closed forms of the full-conditional distributions are not available, we found the joint posterior distributions of model parameters using Markov chain Monte Carlo methods [32], and calculated the posterior distribution of HRs with respect to certain standard intervention (either R-Benda or R-CHOP-R, respectively, as a common comparator). The simulation for posterior distribution was conducted with three different chains, and each of them produced 20,000 iterations with 60,000 burn-in samples and 1/3 thinning rates. The convergence of Markov chains was checked by trace plots and Gelman–Rubin diagnostic statistics. The median of the posterior distribution was selected as the point estimate, bounded by the 2.5th and 97.5th percentiles to form a 95% Bayesian credible interval (95% BCI). Pairwise comparisons of HRs between two treatment arms were summarized in a league table. Furthermore, we estimated the overall ranks of treatments by calculating the surface under the cumulative ranking curve (SUCRA) for each treatment [33]. The SUCRA index ranges between 0 and 1, where the treatments with the highest and lowest SUCRA are considered the most and least efficacious treatments, respectively. We calculated the percentage of simulations in which every candidate treatment ranked first to determine its probability of being the best treatment (PbBT). For each treatment, we also reported the median and 95% BCI of the posterior ranking distribution (PoRa) for all the simulations. In summary, the comparison of candidate treatments was made based on HR, SUCRA, PbBT, and PoRa.

We used statistical software R (version 3.6.2, R project; with packages rjags_v4-10, coda_v0.19-3, lattice_v0.20-38, and ggplot2_v3.3.3) and JAGS (version 4.3.0, http://mcmc-jags.sourceforge.net) for data analysis.

## Results

### Study selection

**Fig. 1:**
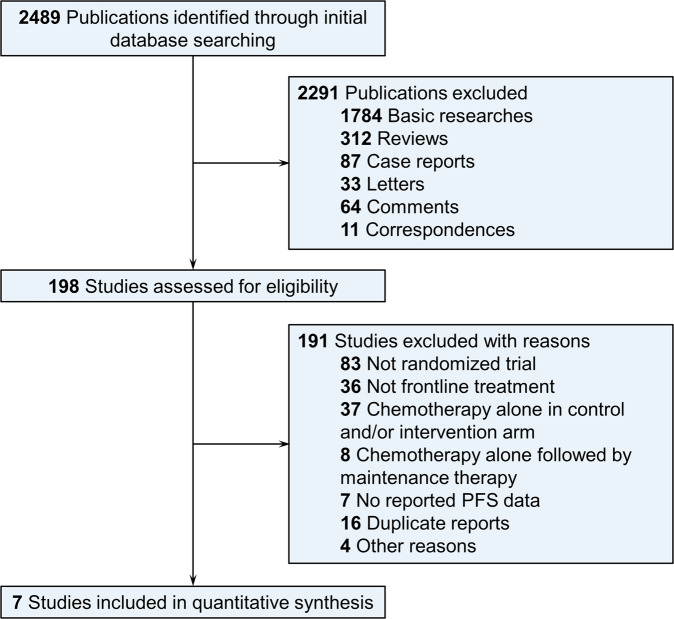
PRISMA flowchart of the study selection process.

The literature search and study selection process are illustrated in Fig. 1. The initial literature search identified 2489 publications. After screening, 2291 non-clinical trial publications, 83 non-randomized clinical trials, and 36 non-front-line trials were excluded. As we were interested in comparing different state-of-the-art immunochemotherapy and maintenance regimens, clinical trials with chemotherapy alone as the comparative arm or comparing maintenance strategies after chemotherapy alone were excluded. In addition, four studies were excluded from the network meta-analysis due to lower relevance: JCOG 0203 (R-CHOP-21 vs R-CHOP-14 for indolent NHL), SWOG S0016 (CHOP-R with non-classical dosing schedule vs CHOP followed by two doses of ^131^I-tositumomab), NCT01144364 (rituximab maintenance vs observation after immunochemotherapy with the R-FND regimen), and SABRINA (subcutaneous vs intravenous rituximab). Finally, a total of seven studies were selected for the network meta-analysis.

### Network composition

**Fig. 2:**
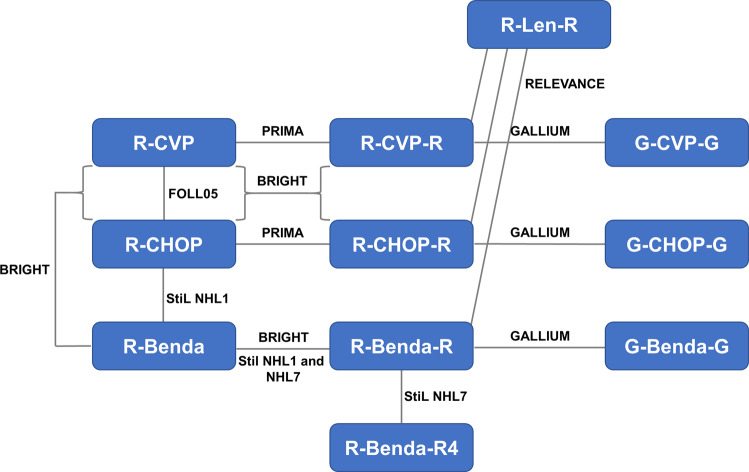
Network of comparisons included in the network meta-analysis.

The seven studies included in the network were FOLL05 [7, 8], PRIMA [9, 10], StiL NHL1 [11], BRIGHT [12–14], StiL NHL7 [15], GALLIUM [16, 17], and RELEVANCE [18]. These randomized phase 3 trials included a total of 4557 participants. Characteristics and efficacy data of the included studies are summarized in Table 1. Figure 2 shows the network of 11 different treatment regimens with different immunochemotherapy and maintenance strategies, including R-CVP, R-CHOP, R-Benda, R-CVP-R, R-CHOP-R, B-Benda-R, R-Benda-R4, G-CVP-G, G-CHOP-G, G-Benda-G, and R-Len-R. Maintenance with rituximab (-R) or obinutuzumab (-G) was administered for up to 2 years, except for R-Benda-R4 which included 4 years of rituximab maintenance.

**Table 1:** Characteristics and PFS data of included randomized controlled trials.

Trial name	Stage	Grade	Trial design	Maintenance	Data source	Arms	Patient number	PFS HR (95% CI)
FOLL05 [7, 8]	II–IV	1, 2, 3a	R-CHOP vs R-CVP vs R-FM Primary endpoint: time to treatment failure	Not allowed	Federico et al. [7]	R-CHOP	165	0.73 (0.54–0.98)^a^ *p* = 0.037
						R-CVP	168	
PRIMA [9, 10]	III–IV	1, 2, 3a	Rituximab maintenance vs observation after responding to R-CHOP, R-CVP or R-FM (R-FM accounted for <3% of patients) Primary endpoint: PFS from randomization (6 months after induction)	Randomized	Bachy et al. [10]	Rituximab maintenance	505	0.61 (0.52–0.73)^e^ *p* < 0.0001
						Observation	513	
						R-CHOP-R	382	0.57 (0.47–0.70)
						R-CHOP	386	
						R-CVP-R	109	0.75 (0.53–1.07)
						R-CVP	113	
Stil NHL1 [11]	III–IV	1, 2	R-Benda vs R-CHOP for iNHL and MCL Primary endpoint: PFS	Not allowed	Rummel et al. [11]	R-Benda	139	0.61 (0.42–0.87)^b^ *p* = 0.0072
						R-CHOP	140	
BRIGHT [12–14]	II–IV	1, 2	R-Benda vs R-CHOP/R-CVP for iNHL and MCL Primary endpoint: CR rate (noninferiority design)	Allowed (at the discretion of the investigator)	Flinn et al. [14]^c^	R-Benda(-R)	187	0.70 (0.49–1.01) *p* = 0.0582
						R-CHOP(-R)/ R-CVP(-R)	186	
					Kahl et al. [13]^d^	R-Benda-R	81	0.50 (0.26–0.94) *p* = 0.0295
						R-Benda	63	
						R-CHOP-R/ R-CVP-R	83	0.66 (0.38–1.16) *p* = 0.1443
						R-CHOP/ R-CVP	61	
Stil NHL7 (MAINTAIN) [15]	II–IV	1, 2	Rituximab maintenance (R4) vs observation (R) after responding to R-Benda and 2 years of rituximab maintenance Primary endpoint: PFS from randomization	Randomized	Rummel et al. [15]	R-Benda-R4	178	0.73 (0.44–1.21) *p* = 0.1125
						R-Benda-R	172	
			Rituximab maintenance (up to 2 years) vs observation after responding to R-Benda (R4 patients censored after 2 years of maintenance) Primary endpoint: PFS	Indirect comparison combining NHL1 and NHL7 (a secondary endpoint of NHL7)	Rummel et al. [15]	R-Benda-R	595	0.68 (0.47–0.87) *p* = 0.0074
						R-Benda	139	
GALLIUM [16, 17]	III–IV	1, 2, 3a	G-Chemo-G vs R-Chemo-R Primary endpoint: PFS	Required (if responding to induction; >90% received maintenance)	Hiddemann et al. [17]	G-Chemo-G	601	0.68 (0.54–0.87)^e^ *p* = 0.0016
						R-Chemo-R	601	
						G-Benda-G	345	0.63 (0.46–0.88) *p* = 0.0062
						R-Benda-R	341	
						G-CHOP-G	196	0.72 (0.48–1.10) *p* = 0.13
						R-CHOP-R	203	
						G-CVP-G	60	0.79 (0.42–1.47) *p* = 0.46
						R-CVP-R	57	
RELEVANCE [18]	II–IV	1, 2, 3a	R-Len-R vs R-Chemo-R Primary endpoint: CR/CRu rate (at 120 weeks) and PFS	Required (if responding to induction; >85% received maintenance)	Morschhauser et al. [18]	R-Len-R	513	1.10 (0.85–1.43)^e^ *p* = 0.48
						R-Chemo-R	517	
						R-Len-R	100	1.75 (0.88–3.49)
						R-Benda-R	117	
						R-Len-R	383	1.02 (0.76–1.37)
						R-CHOP-R	373	
						R-Len-R	100	1.75 (0.88–3.49)
						R-CVP-R	27	

*PFS* progression-free survival, *HR* hazard ratio, *CI* confidence interval, *iNHL* indolent non-Hodgkin lymphoma, *MCL* mantle cell lymphoma, *FL* follicular lymphoma, *LPL* lymphoplasmacytic lymphoma, *MZL* marginal zone lymphoma, *CR* complete response, *CRu* unconfirmed complete response.

^a^PFS data adjusted by Follicular Lymphoma International Prognostic Index 2 (FLIPI2).

^b^PFS data for FL patients only.

^c^Data for the entire iNHL cohort (predominantly FL). R-Benda arm, FL *n* = 154, other iNHL (LPL, MZL) *n* = 33; R-CHOP/R-CVP arm, FL *n* = 160, other iNHL *n* = 26.

^d^Data for FL patients only. Post hoc analysis of (non-randomized) rituximab maintenance vs observation in patients who achieved an objective response.

^e^These data were not used in the network meta-analysis.

### Ranking for regimens based on PFS

**Fig. 3:**
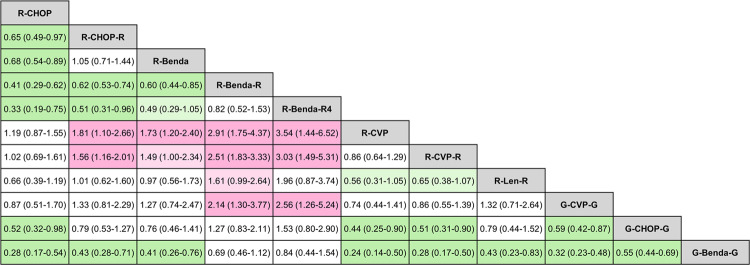
League table of network meta-analysis results. Direct and indirect comparisons of PFS of different regimens were shown. The table should be read from left to right. Hazard ratios for comparisons are in the cell in common between the column-defining and row-defining treatment. A hazard ratio of <1 favors row-defining treatment.

Pairwise comparisons of PFS among all treatment regimens are shown in a league table (Fig. 3). SUCRA, PbBT, and PoRa for each regimen are shown in Table 2.

**Table 2:** Ranking of immunochemotherapy regimens by PFS.

Regimen	SUCRA	PbBT	PoRa [95% BCI]
G-Benda-G	0.97	72%	1 [1–3]
R-Benda-R4	0.88	25%	2 [1–5]
R-Benda-R	0.81	3%	3 [1–4]
G-CHOP-G	0.66	0%	4 [2–7]
R-CHOP-R	0.51	0%	6 [4–8]
R-Len-R	0.5	0%	6 [3–10]
R-Benda	0.48	0%	6 [4–8]
G-CVP-G	0.28	0%	8 [5–11]
R-CHOP	0.19	0%	9 [7–11]
R-CVP-R	0.16	0%	9 [7–11]
R-CVP	0.05	0%	11 [8–11]

*SUCRA* surface under the cumulative ranking curve, *PbBT* probability of being the best treatment, *PoRa* posterior ranking, *BCI* Bayesian credible interval.

G-Benda-G was identified as the regimen that results in the best PFS (HR 0.41 [95% BCI 0.26–0.76] compared to R-Benda; SUCRA 0.97, PbBT 72%, PoRa 1 [95% BCI 1–3]), followed by R-Benda-R4 (HR 0.49 [0.29–1.05] compared to R-Benda; SUCRA 0.88, PbBT 25%, PoRa 2 [1–5]) and R-Benda-R (HR 0.60 [0.44–0.85] compared to R-Benda; SUCRA 0.81, PbBT 3%, PoRa, 3 [1–4]). Different settings of prior distributions of the model were examined and the results were consistent.

### PFS compared to R-Benda and R-CHOP-R

**Fig. 4:**
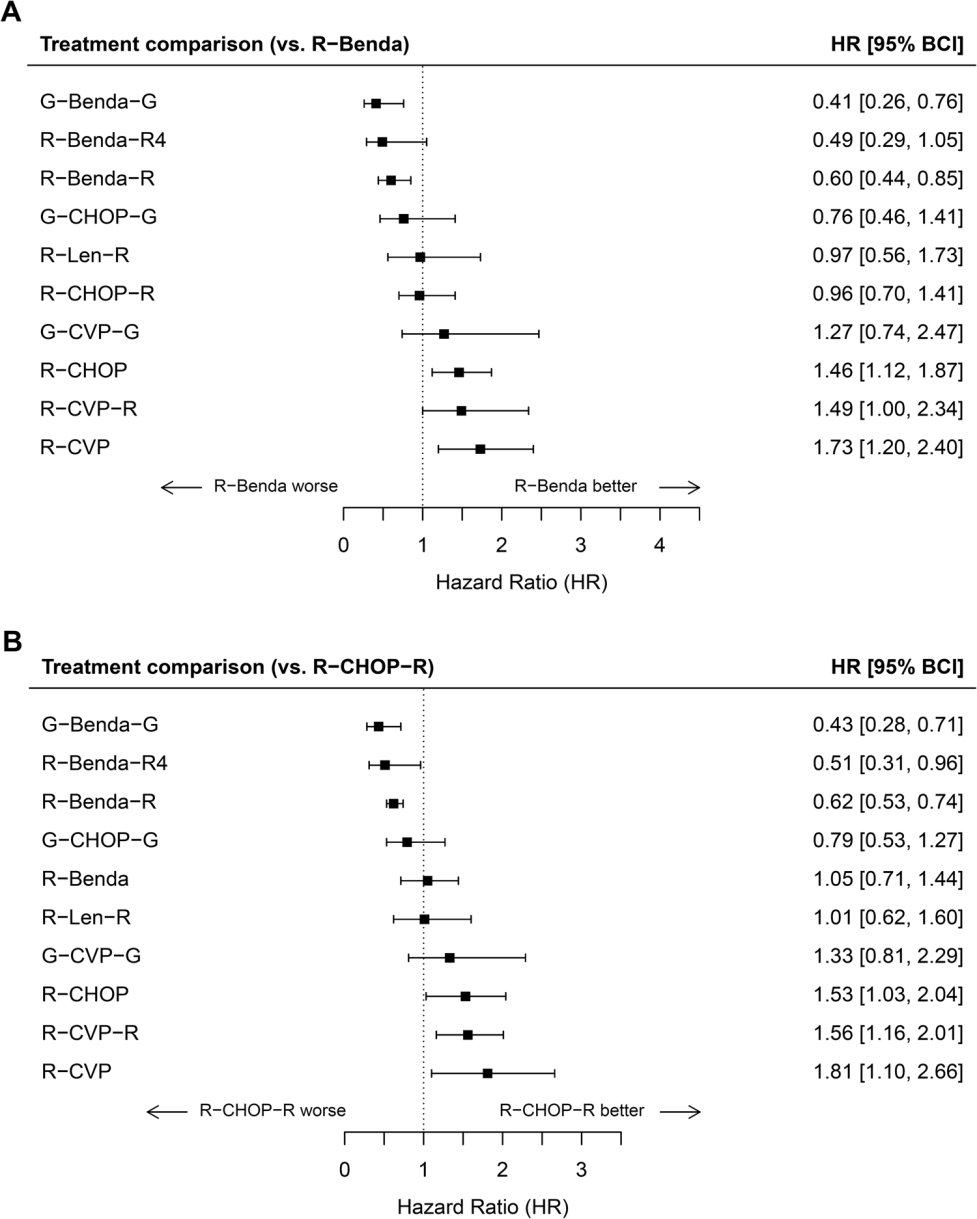
Forest plot of network meta-analysis results. **A** Forest plot of hazard ratios for PFS of other regimens compared to R-Benda. **B** Forest plot of hazard ratios for PFS of other regimens compared to R-CHOP-R.

Comparisons of other regiments with R-Benda, a regimen commonly used in current practice, are shown in Fig. 4A. G-Benda-G, R-Benda-R4, and R-Benda-R were superior to R-Benda, while R-CHOP (HR 1.46 [1.12–1.87]), R-CVP-R (HR 1.49 [1.00–2.34]), and R-CVP (HR 1.73 [1.20–2.40]) were inferior. R-CHOP-R (HR 0.96 [0.70–1.41]) and R-Len-R (HR 0.97 [0.56–1.73]) had very similar efficacy to R-Benda. Comparisons of other regiments with R-CHOP-R are shown in Fig. 4B, and the results were similar to the above.

### Comparisons of backbones and maintenance

Bendamustine was a better backbone therapy than CHOP with consistent improvement in PFS, as indicated either with maintenance (R-Benda-R vs R-CHOP-R, HR 0.62 [0.53–0.74]; G-Benda-G vs G-CHOP-G, HR 0.55 [0.44–0.69]) or without maintenance therapy (R-Benda vs R-CHOP, HR 0.68 [0.54–0.89]). CHOP had similar efficacy to lenalidomide when both were followed by rituximab maintenance (R-Benda-R vs R-CHOP-R, HR 1.01 [0.62–1.60]). CHOP was better than CVP, especially with maintenance therapy (R-CVP-R vs R-CHOP-R, HR 1.56 [1.16–2.01]; G-CVP-G vs G-CHOP-G, HR 1.68 [1.16–2.38]).

Maintenance therapy with rituximab improved PFS following R-CHOP (R-CHOP-R vs R-CHOP, HR 0.65 [0.49–0.97]) or R-Benda (R-Benda-R vs R-Benda, HR 0.60 [0.44–0.85]; R-Benda-R4 vs R-Benda, HR 0.49 [0.29–1.05]). The improvement was not statistically significant following R-CVP (R-CVP-R vs R-CVP, HR 0.86 [0.64–1.29]).

### Sensitivity analysis

The sensitivity Bayesian meta-analyses (i)–(iii) yielded similar relative treatment effects (Supplementary Figs. 1–3) and ranking of treatments (Supplementary Table 1), suggesting the robustness of the reported results. In particular, the G-Benda-G regimen was uniformly ranked the most efficacious regimen with a strong SUCRA score above 0.95 in all models.

## Discussion

FL is heterogenous biologically and clinically, and there is no established single standard-of-care front-line immunochemotherapy regimen for advanced FL. The current NCCN guidelines on B-cell lymphomas (v5.2021) endorse bendamustine, CHOP, CVP in combination with rituximab or obinutuzumab, as well as R-Len, as preferred immunochemotherapy regimens [19]. The guidelines also support optional maintenance with rituximab or obinutuzumab. In this context, our network meta-analysis is the first study to comprehensively compare the efficacy of 11 modern immunochemotherapy regimens for front-line treatment of advanced FL. The analysis was performed in a Bayesian hierarchical modeling framework to incorporate both direct and indirect evidences, and the assessments included pairwise comparisons as well as ranking of different treatment regimens. Results of this meta-analysis provide an important reference for clinicians to choose between available front-line regimens, especially when a better PFS is desired.

Clinical decision is a complicated process. Efficacy, side effects, comorbidities, patient preferences and logistics all need to be taken into consideration. Therefore, this study is not meant to identify one regimen as the most efficacious that clinicians should use for all patients. Rather, the goal is to synthesize PFS data from large RCTs to make efficacy evidences available to clinicians. The pairwise efficacy comparisons and ranking of available regimens can help clinicians better weigh the potential benefits and risks when trying to choose the best treatment regimen for any given patient. For example, while R-Benda appears to be the most popular regimen in current clinical practice, choosing regimens that are associated with a better PFS would be reasonable in young, fit patients with high-risk diseases (e.g., high-risk FLIPI or high-risk for POD24), for example, G-Benda-G, R-Benda-R4, and R-Benda-R based on our study. Importantly, that is not to say, “the more, the better”. Our study focused solely on PFS data. A similar network analysis on OS was not completed, because (1) a network could not be constructed due to lack of complete data, and (2) OS was not the primary endpoint of the included studies. In addition, a detailed comparison of adverse events (AEs) between the regimens was not completed due to insufficient data on regimen-specific AE data. These endpoints should be considered carefully when selecting regimens for specific patients. For example, for high-risk patients, while regimens such as G-Benda-G, R-Benda-R4, and R-Benda-R are associated with better PFS and are attractive, it is important to recognize that there is no evidence of improved OS with these regimens compared to R-Benda, and longer and more immunosuppressive treatments may be detrimental in specific situations, for example, in the current COVID-19 pandemic era.

Our study demonstrated that bendamustine is more efficacious than CHOP, as supported by the superior PFS with R-Benda vs R-CHOP, R-Benda-R vs R-CHOP-R, and G-Benda-G vs G-CHOP-G. The better PFS and more favorable tolerability during therapy make bendamustine a preferred chemotherapy backbone. Choosing bendamustine over CHOP also allows reserving CHOP for transformation, which fares better in the anthracycline-naïve setting [34]. However, several caveats should be noted. First, bendamustine can cause more lymphopenia (which can be prolonged), increased second malignancies, and possibly increased mortality in older patients (>70 years old) compared to CHOP [11, 12, 16, 17]. With long-term follow-up, 37 of 215 patients treated with R-Benda (compared to 40 of 205 with R-CHOP) in StiL NHL1 trial and 42 of 221 patients treated with R-Benda (compared to 24 of 215 with R-CHOP/R-CVP, *p* = 0.022) in BRIGHT trial developed second malignancies. Second, the StiL NHL1 and BRIGHT trials only included grade 1–2 FL [11, 12], and there is limited data supporting the use of bendamustine in grade 3A FL. Therefore, despite of increasing use of R-Benda (with or without rituximab maintenance) in current clinical practice, CHOP is still appropriate for select patients, e.g., grade 3A FL. In addition, patients with a relatively high SUV (e.g., ≥13–18) on PET scan may benefit from CHOP vs bendamustine [35, 36].

For the comparison between obinutuzumab and rituximab, only direct evidences were available (from GALLIUM trial) for this network meta-analysis. The estimated HRs in our model were slightly different from those reported in the GALLIUM subgroup analysis. Due to the Bayesian shrinkage estimation from a common variance parameter for treatment, the applied hierarchical structure retains the same relative ranking but delivers more conservative comparisons of treatment effects between various regimens. For example, for G-Benda-G vs R-Benda-R, the HR was 0.69 (95% BCI 0.46–1.12) in our model vs 0.63 (95% CI 0.46–0.88) in GALLIUM. When combined with the same backbone, obinutuzumab-based therapy always ranked higher than rituximab-based therapy in our study, consistent with GALLIUM. Notably, G-Benda-G was ranked as the most efficacious regimen based on PFS, with a strong SUCRA score of 0.97 and a probability of 72% as the most efficacious. This regimen can be considered in high-risk patients when feasible. The risk of increased side effects with obinutuzumab needs to be weighed carefully [16].

Regarding maintenance therapy, our study showed that rituximab maintenance improved PFS following R-CHOP but not R-CVP. Our findings further support the data of BRIGHT and PRIMA, which were inadequately powered for subgroup comparisons. A retrospective multicenter study suggested a PFS benefit of rituximab maintenance following R-Benda, although the benefit was only statistically significant in patients who achieved a partial response to induction, but not those with a complete response [37]. However, there has been no RCT comparing rituximab maintenance vs observation following R-Benda. The Stil NHL7 trial only compares 2 additional years of maintenance vs observation following R-Benda and 2 years of rituximab maintenance, i.e., 4 years vs 2 years of maintenance. Incorporating indirect comparisons across RCTs, our study showed that 2 years of rituximab maintenance significantly increases PFS following R-Benda (HR 0.60, 95% BCI 0.44–0.85). Four years of maintenance likely further improves PFS (HR 0.49, 95% BCI 0.29–1.05), but the robustness of this conclusion is limited by a smaller patient number in the R-Benda-R4 group. While our data support the use of rituximab maintenance following R-CHOP and R-Benda but not R-CVP, many factors need to be considered to make individualized decisions, including response to the induction immunochemotherapy, potential side effects with long-term use, comorbidities, etc. On the other hand, obinutuzumab maintenance was uniformly given following obinutuzumab-containing immunochemotherapy in the GALLIUM trial, although the benefit of obinutuzumab maintenance vs observation is completely unknown. Of note, NCCN guidelines accept obinutuzumab-containing immunochemotherapy without obinutuzumab maintenance as one of the preferred options, acknowledging that “the use without maintenance was an extrapolation of the data” [19].

The strengths of this study include a comprehensive systematic review of literature for study selection, inclusion of large phase 3 RCTs, and successful construction of a network to compare 11 modern immunochemotherapy regimens. In addition, we applied flexible Bayesian hierarchical modeling, with incorporation of both direct and indirect evidences, to “borrow strength” across trials and shrinkage estimation to derive conservative inference and conclusions. Sensitivity analyses with different model prior specifications and inclusion criteria highlighted the robustness of the results.

This study has several limitations. First, like every other meta-analysis, there is some heterogeneity in the design and reporting of the included trials. FL grade, stage, and treatment indications were not uniform. In addition, maintenance allowance differed between the included studies (Table 1), which could affect the transitivity assumption. We accounted for some of the heterogeneity through sensitivity analyses (i)–(iii) and did not observe any notable changes in our findings. Second, only phase 3 RCTs were included in this network meta-analysis. While phase 3 trials usually are well designed and implemented to deliver high-quality clinical outcome data, the total number of studies could have been limited. Though the intervention network (Fig. 2) was successfully connected, most direct comparisons were informed by only a single RCT connection. The relatively sparse direct evidence for pairwise comparison limited incoherence assessment. Third, this meta-analysis was conducted based on study-level data. Individual patient characteristics may have substantial influences on treatment outcomes, which deserves further studies in the future. Fourth, data from subgroup analysis or post hoc analysis was used for some trials, which were not powered adequately in the studies. Last but not least, with PFS being the primary efficacy endpoint in this study, OS and AE data were not compared between regimens, partly due to the lack of subgroup data in included studies. Knowing both the efficacy and potential short- and long-term AEs, clinicians will make better decisions for their patients.

In conclusion, our study found that for treatment-naïve advanced FL, G-Benda-G had the highest efficacy as measured by PFS. In addition, bendamustine was superior to CHOP as a chemotherapy backbone, and rituximab maintenance following R-Benda improved PFS. In the absence of multi-arm RCTs that include all common regimens with various immunochemotherapy and maintenance strategies, this study provides an important and useful guide to inform treatment decisions.

## Supplementary information

Supplemental material

Checklist
