# Supplementary material for: Efficacy of front-line immunochemotherapy for follicular lymphoma: a network meta-analysis of randomized controlled trials
Source: Blood Cancer J. 2022 Jan 5;12(1):1. doi: 10.1038/s41408-021-00598-x (PMC8728708; doi:10.1038/s41408-021-00598-x)
Supplement: Supplementary file 1 — Supplemental material [file 41408_2021_598_MOESM1_ESM.docx]

**Supplemental material**

**Supplementary Table 1. Ranking of immunochemotherapy regimens in sensitivity analyses**

| Regimen | SUCRA | | | PbBT | | | PoRa [95% BCI] | | |
| --- | --- | --- | --- | --- | --- | --- | --- | --- | --- |
|  | (i) | (ii) | (iii) | (i) | (ii) | (iii) | (i) | (ii) | (iii) |
| G-Benda-G | 0.96 | 0.95 | 0.96 | 71% | 67% | 71% | 1 [1-3] | 1 [1-4] | 1 [1-4] |
| R-Benda-R4 | 0.88 | 0.82 | 0.85 | 25% | 23% | 21% | 2 [1-5] | 2 [1-8] | 2 [1-7] |
| R-Benda-R | 0.81 | 0.82 | 0.8 | 3% | 6% | 4% | 3 [1-4] | 3 [1-5] | 3 [1-5] |
| G-CHOP-G | 0.66 | 0.61 | 0.63 | 0% | 0% | 0% | 4 [2-7] | 4 [2-9] | 4 [2-9] |
| R-CHOP-R | 0.53 | 0.49 | 0.49 | 0% | 0% | 0% | 6 [4-8] | 6 [4-9] | 6 [4-9] |
| R-Len-R | 0.51 | 0.49 | 0.49 | 0% | 1% | 1% | 6 [3-10] | 6 [2-11] | 6 [2-10] |
| R-Benda | 0.44 | 0.52 | 0.54 | 0% | 3% | 3% | 7 [4-9] | 6 [1-10] | 6 [1-9] |
| G-CVP-G | 0.29 | 0.24 | 0.25 | 0% | 0% | 0% | 8 [5-11] | 9 [5-11] | 8 [5-11] |
| R-CHOP | 0.17 | 0.27 | 0.24 | 0% | 0% | 0% | 10 [7-11] | 9 [3-11] | 9 [4-11] |
| R-CVP-R | 0.18 | 0.15 | 0.15 | 0% | 0% | 0% | 9 [7-11] | 10 [7-11] | 10 [7-11] |
| R-CVP | 0.05 | 0.14 | 0.09 | 0% | 0% | 0% | 11 [8-11] | 10 [5-11] | 11 [6-11] |

(i). excluding the data on the treatment effect estimation between R-Benda and R-Benda-R from the cross-trial comparison in Stil NHL1 and NHL7 studies;

(ii). from (i), further excluding the data on the post hoc assessment of treatment effects for maintenance therapy in the BRIGHT trial;

(iii). from (ii), further excluding the treatment effect estimation with the randomized but combined control group data in the BRIGHT study.

Abbreviations: SUCRA, surface under the cumulative ranking curve; PbBT, probability of being the best treatment; PoRa, posterior ranking; BCI, Bayesian credible interval.

**Supplementary Figure 1.** **Forest plot of sensitivity analysis (i).** (A) Forest plot of hazard ratios for PFS of other regimens compared to R-Benda. (B) Forest plot of hazard ratios for PFS of other regimens compared to R-CHOP-R.

**A.**

**B.**

**Supplementary Figure 2.** **Forest plot of sensitivity analysis (ii).** (A) Forest plot of hazard ratios for PFS of other regimens compared to R-Benda. (B) Forest plot of hazard ratios for PFS of other regimens compared to R-CHOP-R.

**A.**

**B.**

**Supplementary Figure 3.** **Forest plot of sensitivity analysis (iii).** (A) Forest plot of hazard ratios for PFS of other regimens compared to R-Benda. (B) Forest plot of hazard ratios for PFS of other regimens compared to R-CHOP-R.

**A.**

**B.**
